# Simultaneous Seroprevalence to *Toxoplasma gondii*, Cytomegalovirus and Rubella Virus in Childbearing Women from Western Romania

**DOI:** 10.3390/medicina57090927

**Published:** 2021-09-02

**Authors:** Adelina Geanina Mocanu, Florin Gorun, Ioana Ciohat, Dan Navolan, Daniel Malita, Tatjana Vilibic-Cavlek, George Dahma, Radu Neamtu, Daniela Popescu, Andreea Cioca, Marius Craina

**Affiliations:** 1 Department of Obstetrics-Gynecology and Neonatology, “Victor Babes” University of Medicine and Pharmacy, 300041 Timisoara, Romania; adelina.erimescu@umft.ro (A.G.M.); gorun.florin@umft.ro (F.G.); george.dahma@umft.ro (G.D.); neamtu.radu@umft.ro (R.N.); popescu.daniela@umft.ro (D.P.); craina.marius@umft.ro (M.C.); 2 Laboratory of Antenatal Medicine, Emergency Clinical City Hospital, 300041 Timisoara, Romania; ioana.ciohat@umft.ro; 3 Department of Radiology, “Victor Babes” University of Medicine and Pharmacy, 300041 Timisoara, Romania; 4 Department of Virology, Croatian Institute of Public Health, School of Medicine, University of Zagreb, 10000 Zagreb, Croatia; tatjana.vilibic-cavlek@hzjz.hr; 5 Department of Pathology, Premiere Hospital, 300643 Timisoara, Romania; cioca_andre@yahoo.com

**Keywords:** TORCH complex, *Toxoplasma gondii*, cytomegalovirus, rubella virus, seroprevalence, susceptibility, congenital infection, demography

## Abstract

*Background and Objectives*: *Toxoplasma gondii*, cytomegalovirus (CMV) and rubella virus, besides other agents, belong to a group named the TORCH complex. Research on the epidemiology of these agents in women is of particular interest, as primary infection during pregnancy could cause severe damage to the fetus. Women who had contracted infection before pregnancy develop IgG antibodies, so the fetus is protected in case of contact with the same agent. Our scope was to identify the childbearing women simultaneously protected or susceptible to a primary infection to two or three agents mentioned above. *Materials and Methods:* A cross-sectional study was performed on 6961 fertile Caucasian women from Western Romania, to analyze the simultaneous seroprevalence to two or three of the pathogens from the TORCH complex: *Toxoplasma gondii*, CMV, and rubella virus. Sampling was conducted at two time points: 2008–2010 (group 1; 1461 participants) and 2015–2018 (group 2; 5500 participants). *Results*: The percentage of women simultaneously seropositive to IgG-anti-*Toxoplasma gondii*/IgG-anti-CMV, IgG-anti-*Toxoplasma gondii*/IgG-anti-rubella, IgG-anti-CMV/IgG-anti-rubella or IgG-anti-*Toxoplasma gondii* and IgG-anti-CMV/IgG-anti-rubella antibodies decreased between the two groups (2008–2010 vs. 2015–2018): 41.4% vs. 36.1%, OR = 0.79, *p* = 0.0002; 41.8% vs. 35.7%, OR = 0.77, *p* < 0.0001; 88.9% vs. 83.6%, OR = 0.63, *p* < 0.0001; 39.6% vs. 33.2%, OR = 0.75, *p* < 0.0001. When comparing women from urban and rural areas, the simultaneous seroprevalence was higher in rural areas. In women tested 2008–2010 (group 1) the simultaneous seroprevalence (urban vs. rural) was: 38.4% vs. 49.1%, OR = 1.54, *p* = 0.0002; 38.4% vs. 50.6%, OR = 1.64, *p* < 0.0001; 88.8% vs. 89.2%, OR = 1.04, NS; 36.4% vs. 47.7%, OR = 1.58, *p* = 0.0001. A similar trend was found in women tested in group 2. *Conclusions*: The rate of simultaneous seropositivity to *Toxoplasma gondii*, CMV and rubella virus among Romanian women of reproductive age decreased significantly between 2008–2010 and 2015–2018 and the susceptibility to infections increased. It is necessary to apply increased prevention measures among susceptible pregnant women.

## 1. Introduction

The TORCH complex refers to a group of infectious agents which include *Toxoplasma gondii*, Others (syphilis, hepatitis B, HIV, varicella-zoster virus, parvovirus B19, etc.), rubella virus, cytomegalovirus (CMV) and herpes simplex viruses (HSV type 1 and 2) [1]. *Toxoplasma gondii*, the causative agent of toxoplasmosis, is an obligate intracellular protozoan that infects warm-blooded animals including humans (2). *Toxoplasma gondii* is spread globally, approximately one-third of people around the world are infected with latent toxoplasmosis [2,3]. Congenital toxoplasmosis could appear when a pregnant woman is infected for the first time, or if reactivation of a latent infection occurs, mainly in immunosuppressed persons [2]. The classic triad of congenital toxoplasmosis includes chorioretinitis, hydrocephalus and intracranial calcifications [4]. Microcephaly, hepatosplenomegaly, jaundice, maculopapular or petechial rash, myocarditis, pneumonitis and respiratory distress, convulsions,.hearing defects, an erythroblastosis-like picture, thrombocytopenia, lymphocytosis, monocytosis, and nephrotic syndrome are other occasional findings [5]. CMV is an enveloped, double-stranded DNA virus belonging to the herpesvirus family [5,6]. With an overall birth prevalence of approximately 0.6%, congenital CMV is the most common congenital viral infection in the developed world [7]. CMV is mostly asymptomatic or mildly symptomatic in infants, children and adults [7]. However, congenital infection can be devastating including neurological sequelae. Clinical findings of congenital infection may include intrauterine growth restriction, fetal hydrops, generalized petechiae, purpura, thrombocytopenia, jaundice, hepatosplenomegaly, pneumonitis, microcephaly, periventricular calcifications, seizures, chorioretinitis, sensorineural hearing loss, bone abnormalities, abnormal dentition, and hypocalcified enamel. The most frequent sequel is sensorineural hearing loss [7]. Congenital CMV infection is the most common cause of congenital, non-genetic sensorineural hearing loss in the USA with approximately 40,000 infected children per year [6]. The causative agent of rubella is the rubella virus, an enveloped, single-stranded, positive-sense RNA virus, belonging to the family *Togaviridae* and the sole member of the genus *Rubivirus* [8]. Rubella is usually a mild, self-limited disease associated with a characteristic rash. Rubella virus infection during pregnancy may cause congenital rubella syndrome (CRS), spontaneous abortion and stillbirth [9]. The classic triad, especially when the infection occurs during embryogenesis, is represented by cataracts, heart abnormalities and sensorineural deafness [10]. However, many other transient, permanent or late-onset defects might be observed. Permanent defects include microphthalmia, cataracts, retinopathy, sensorineural deafness, patent ductus arteriosus, pulmonary artery hypoplasia, mental or psychomotor retardation, language delay and microcephaly [10]. The most common defect is deafness, and it may be the only defect observed, especially in cases where the infection occurs between 12 and 18 weeks of pregnancy [10]. Since infections caused by *Toxoplasma gondii*, CMV and rubella virus determine often unspecific or only subclinical forms of disease, it is possible to evolve unobserved in pregnant women. Depending on the time when infection occurs during pregnancy, the lesions in the offspring could occur before or after birth. Usually, the earlier the infection appears, the more serious the lesions are. Instead, transmission is more likely if the infection occurs later in pregnancy. Usually, only not immunized women are at risk of vertically transmitting the pathogen and carrying an affected fetus following the infection. Certain exceptions exist, such as in immunocompromised women or when a new infection with another strain occurs. Public health systems have two strategies to deal with TORCH infections in pregnancy. The first involves early pregnancy testing of immunological status against TORCH agents in women. The second consists in detecting ultrasound signs of fetal infection followed by diagnostic tests. The choice of strategy depends on public health authorities, which decide, based on data including the percentage of women immunized against TORCH agents or coverage, with physicians and medical services. Most studies analyze the seroprevalence of a single pathogen in pregnant women while only a few report on the simultaneous immunization against two or three pathogenic agents. The aim of this study was to identify the childbearing women who are simultaneously protected or susceptible to a primary infection to two or three pathogenic agents that belong to TORCH complex, and also to analyze the changes of these parameters along a period of ten years according to the medium of residence.

## 2. Materials and Methods

### 2.1. Study Design

A cross-sectional study was performed on 6961 fertile women from Western Romania enrolled according to a consecutive case population base, to analyze the simultaneous immunization rate to two or three of the pathogens from the TORCH complex: *Toxoplasma gondii*, CMV, and rubella virus.

### 2.2. Setting

Each participant was tested for the presence of IgG and IgM antibodies against *Toxoplasma gondii*, CMV and rubella virus. Serum samples were collected from fertile women in two different periods: 1461 samples were collected in the period 2008–2010 at the Municipal Hospital Timisoara (group 1) and 5500 samples were collected in the period 2015–2018 at BIOCLINICA SA (private laboratory) (group 2).

### 2.3. Participants

The participants included in our study were Caucasian women of childbearing age, residing in one of the 5 counties (Timis, Caras-Severin, Arad, Hunedoara and Mehedinti) in western Romania who have undergone TORCH screening voluntarily.

### 2.4. Laboratory Analysis

IgG- and IgM-anti-*Toxoplasma gondii*, anti-CMV and anti-rubella antibody titers were determined by chemiluminescence (CLIA) method, using an Immulite One Machine (DPC, Diagnostic Products Corporation, Los Angeles, USA) for the participants tested in 2008–2010 (group 1). In group 2 patients (2015–2018) IgG- and IgM-anti-*Toxoplasma gondii,* respectively, IgG- and IgM-anti-rubella virus antibodies titers were measured by chemiluminescent microparticle immunoassay (CMIA) method using an Architect i1000SR engine (Abbott Park, Illinois, Chicago, IL, USA) and commercial tests (Abbott, Max-Planck-Ring 2, Wiesbaden, Germany). Additionally, IgG- and IgM-anti-CMV antibodies were tested using an Immulite 2000 Machine (Diagnostic Products Corporation, Los Angeles, CA, USA) and commercial tests (Siemens Healthcare Diagnostics Products, Llanberis, Gwynedd, United Kingdom) A cut-off (CO) was calculated for each type of assay. The calculations of the results were performed according to the following formula: sample cps (counts per second)/CO. Values above CO were considered positive, and respectively, values below CO were considered negative.

### 2.5. Statistical Methods

The values of IgG- and IgM- antibody titers against the three pathogens were stored besides demographic data in Astraia software (Astraia GmbH, Munich, Germany) (group 1-2008–2010) or Microsoft Office Excel 2003 (Microsoft Co, Redmond, WA, USA) (group 2-2015–2018). Statistical analysis was performed using Instat Prisma 8.0.2 (GraphPad Software, San Diego, CA, USA). Fisher’s exact test was used to compare proportions. *p* < 0.05 was considered significant. Data were represented in median and interquartile intervals.

### 2.6. Ethical Issues

The study was approved by the Institutional Board of the “Victor Babes” University of Medicine and Pharmacy (Timisoara, Romania) approval no. 848 from 6 April 2011. Informed consent was obtained from each patient.

## 3. Results

### 3.1. Demographic Features of Participants

The demographic features of the 1461 women included in group 1 (2008–2010) and 5500 women included in group 2 (tested 2015–2018) are presented in Table 1

### 3.2. Prevalence of IgG and IgM Antibodies against a Single Pathogen: 2008–2010 vs. 2015–2018

A statistically significant decrease was found between the two groups of women (2008–2010 vs. 2015–2018) regarding Toxoplasma gondii- (43.7% vs. 38.8%, *p* = 0.0006), CMV- (94.7% vs. 91.1%, *p* < 0.0001), respectively, rubella virus- IgG seroprevalence (94.1% vs. 91.5%, *p* = 0.0009). No statistically significant difference was found between the two studied groups (2008–2010 vs. 2015–2018) regarding IgM anti- Toxoplasma gondii (0.8% vs. 1.1%, NS), -CMV (0.3% vs. 0.35, NS) or -rubella virus seroprevalence (0.5% vs. 0.3%, NS) (Table 2).

IgG and IgM anti-*Toxoplasma gondii*, anti-CMV and anti-rubella virus antibodies were present simultaneously in 0.8%, 0.3% and 0.5% sera of women from group 1 (2008–2010), respectively, and 1.0%, 0.3% and 0.3% sera of women from group 2 (2015–2018) (Table 3).

### 3.3. Simultaneous IgG Seroprevalence against Two or Three Pathogens in Women from Urban and Rural Areas: 2008–2010 vs. 2015–2018

The rate of women at childbearing age simultaneously seropositive to IgG-anti-*Toxoplasma gondii*/IgG-anti-CMV, IgG-anti-*Toxoplasma gondii*/IgG-anti-rubella, IgG-anti-CMV/IgG-anti-rubella or IgG-anti-*Toxoplasma gondii* and IgG-anti-CMV/IgG-anti-rubella antibodies decreased between the two groups (2008–2010 vs. 2015–2018): 41.4% vs. 36.1%, OR = 0.79, *p* = 0.0002; 41.8% vs. 35.7%, OR = 0.77, *p* < 0.0001; 88.9% vs. 83.6%, OR = 0.63, *p* < 0.0001; 39.6% vs. 33.2%, OR = 0.75, *p* < 0.0001. A similar trend was found in urban areas: 38.4% vs. 31.8%, OR = 0.74, *p* < 0.0001; 38.4% vs. 32.2%, OR = 0.76, *p* = 0.0002; 88.8% vs. 81.9%, OR = 0.57, *p* < 0.0001; 36.4% vs. 29.4%, OR = 0.72, *p* < 0.0001. The negative trend along the last decade was present in the rural areas too, but it reaches a statistically significant threshold only in certain categories: 49.1% vs. 44.1%, OR = 0.81, *p* = 0.07; 50.6% vs. 42.1%, OR = 0.71, *p* = 0.002; 89.2% vs. 86.6%, OR = 0.78, *p* = NS; 47.7% vs. 40.2%, OR = 0.73, *p* = 0.005 (Table 4).

Comparing women from urban and rural areas, the simultaneous seroprevalence to IgG-anti-*Toxoplasma gondii*/IgG-anti-CMV, IgG-anti-*Toxoplasma gondii*/IgG-anti-rubella, IgG-anti-CMV/IgG-anti-rubella or IgG-anti-*Toxoplasma gondii* and IgG-anti-CMV/IgG-anti-rubella antibodies was higher in rural areas. In women tested 2008–2010 (group 1) the simultaneous seroprevalence was (urban vs. rural): 38.4% vs. 49.1%, OR = 1.54, *p* = 0.0002; 38.4% vs. 50.6%, OR = 1.64, *p* < 0.0001; 88.8% vs. 89.2%, OR = 1.04, NS; 36.4% vs. 47.7%, OR = 1.58, *p* = 0.0001. In women tested 2015–2018 (group 2) the simultaneous seroprevalence was (urban vs. rural): 31.8% vs. 44.1%, OR = 1.69, *p* < 0.0001; 32.2% vs. 42.1%, OR = 1.53, *p* < 0.0001; 81.9% vs. 86.6%, OR = 1.42, *p* < 0.0001; 29.4% vs. 40.2%, OR = 1.61, *p* < 0.0001 (Table 4).

### 3.4. Simultaneous Susceptibility to Two or Three Pathogens in Women from Urban and Rural Areas: 2008–2010 vs. 2015–2018

Simultaneous susceptibility of women has increased over time from the group tested 2008–2010 to the ones tested 2015–2018, regarding infections with *Toxoplasma gondii*/CMV, *Toxoplasma gondii*/rubella virus, CMV/rubella virus or *Toxoplasma gondii* and CMV/rubella virus (2008–2010 vs. 2015–2018): 2.9% vs. 6.1%, OR = 2.14, *p* < 0.0001; 4.0% vs. 5.3%, OR = 1.35, *p* = 0.003; 0.1% vs. 1.0%, OR = 14.57, *p* < 0.0001; 0% vs. 0.7%, OR = NA, *p* = NA. This increasing trend was found in women from urban areas: 3.4% vs. 7.8%, OR = 2.40, *p* < 0.0001; 3.7% vs. 5.6%, OR = 1.55, *p* = 0.01; 0.1% vs. 1.1%, OR = 12.23, *p* = 0.0003; 0% vs. 0.9%, OR = NA, *p* = NA. In rural areas the increase in susceptibility along the last decade did not reach a statistically significant value: 1.7% vs. 2.9%, OR = 1.7, *p* = NS; 4.7% vs. 4.7%, OR = 1.01, *p* = NS; 0% vs. 0.6%, OR = NA, *p* = NA; 0% vs. 0.25%%, OR = NA, *p* = NA (Table 4).

The comparison between urban and rural areas showed different rates of simultaneous susceptibility to *Toxoplasma gondii*/CMV, *Toxoplasma gondii*/rubella virus, CMV/rubella virus or *Toxoplasma gondii,* -CMV/rubella virus: 3.4% vs.1.7% OR = 0.49, *p* = NS), 3.7% vs. 4.7% (OR = 1.27, *p* = NS), 0.1% vs. 0%, OR = NA, *p* = NA); 0% vs.0%, OR= NS, *p* = NS) (group 1), respectively, 7.8% vs. 2.9%, OR= 0.35, *p* < 0.0001; 5.6% vs. 4.7%, OR = 0.82, *p* = NS; 1.1% vs. 0.6%, OR= 0.53, *p* = 0.06; 0.9% vs. 0.2%, OR= 0.25, *p* = 0.0003 (group 2) (Table 4).

## 4. Discussion

This study is part of a larger project that demonstrated a decrease in the seroprevalence of *Toxoplasma gondii*, CMV and rubella virus, among women of reproductive age and pregnant women in western Romania over a decade from 2008 to 2018 [11,12,13]. Thus, our previous research showed a decrease in seroprevalence among women of reproductive age, between 2008–2010 and 2015–2018, from 43.8% to 38.8% [11], in the case of *Toxoplasma gondii*. Among the general population, the seroprevalence of *Toxoplasma gondii* is estimated at 25–30%, while among women of reproductive age, it varies between regions being 9.1% in the USA, 19% in Italy and 44% in France. Therefore, it is considered that the western region of Romania is an area with a moderate risk of *Toxoplasma gondii* infection. Among the risk factors associated with *T. gondii* seropositivity, the following are mentioned: the place of residence from the rural environment, the lower socioeconomic level, as well as the more frequent contact with the infected animals and soil. [11]. In the western region of Romania, our previous research showed also a decrease in CMV seroprevalence among women of reproductive age, from 94% to 91.8% [12]. An increased seroprevalence was found in women from rural compared to urban areas. An explanation for this fact could be the mode of CMV transmission, low socio-economic status and poor hygienic measures as risk factors for infection [14]. Latent CMV infection is considered a health risk factor, and co-infections with this virus increase patient morbidity and mortality [15]. Previous data published by our group showed that the seroprevalence of IgG-antibodies against rubella virus decreased from 2008 to 2018 from 94.1% to 91.4% [13]. The main cause for this trend could be as noted: inconsistency in the implementation of the national vaccination program and the refusal of certain categories of people to participate in vaccination programs [13].

Although we included in our research women with positive IgM antibody titers, our results confirm, with small differences, the decreasing trend in seroprevalence found in our previous studies, where IgM positive cases were excluded from statistic evaluation. Furthermore, including women with positive IgM antibody titers gave us the possibility to analyze changes in the prevalence of patients with both IgG and IgM positive titers along this decade. Usually, a positive titer of IgM antibodies is interpreted as suggestive of acute infection [16]. However, in certain cases, IgM antibodies are still detectable years after infection and do not really represent a sign of acute infection [17]. In our study, no statistically significant differences were found regarding the prevalence of cases with simultaneous IgM and IgG antibody titers against *Toxoplasma gondii*, CMV and rubella virus, respectively, between the two groups: 2008–2010 and 2015–2018. Some studies analyzed the seroprevalence of IgM antibodies relative to the seroprevalence of IgG antibodies [14,18,19]. The prevalence of women with IgM positive antibodies titer in our study was lower compared to studies from other countries [18,19]. A study published in Turkey in 2011 showed an IgM-anti-*Toxoplasma gondii* antibodies seroprevalence of 1.34% whereas the seroprevalence of IgG-anti-*Toxoplasma gondii* antibodies was 24.6% [18]. In comparison, the IgM-anti-*Toxoplasma gondii* seroprevalence in our study was in the first group 0.8% with an IgG-anti-*Toxoplasma gondii* seroprevalence of 43.7%, respectively, in the second group 1.1% (IgM) with a seroprevalence of 38.8% (IgG). A study from Croatia reported a seroprevalence of IgM anti-*Toxoplasma gondii* antibodies of 2.4% at an IgG-anti-*Toxoplasma gondii* seroprevalence of 29.1% [19]. In the same study from Croatia, the IgM-CMV seroprevalence was 2.2% at an IgG seroprevalence of 75.3%, whereas the IgM-anti-rubella seroprevalence was 3.2% at an IgG-seroprevalence of 94.6% [19]. In our study, the IgM-CMV seroprevalence was 0.3% for both groups at an IgG seroprevalence of 94.7% (group 1), respectively, of 91.1% (group 2) since the seroprevalence for IgM-anti-rubella virus was 0.5% at an IgG-anti-rubella seroprevalence of 94.1% (group 1), respectively, 0.3% at an IgG-anti-rubella virus seroprevalence of 91.5% (group 2). Although a difference regarding the IgM seroprevalence in our studies exists, this could be explained by the fact that in a Croatian study all the IgM-positive cases showed high IgG avidity, which excludes the presence of acute infection [19].

We studied the immunity status against TORCH agents in many women and this gave us the chance to study both the rate of women protected, respectively, simultaneously susceptible against two or three pathogens, and to analyze these parameters according to the place of provenience. In addition, we studied changes of these parameters between the two groups of women tested in 2008–2010 and 2015–2018, respectively.

Compared to urban areas, women from rural areas showed higher simultaneous seroprevalence to two or all three pathogens in both groups 2008–2010 and 2015–2018, except the simultaneous seroprevalence to CMV and rubella virus where the difference did not reach a statistically significant threshold. An explanation could be that both pathogens (CMV and rubella virus) had a very high seroprevalence in women of childbearing age. Regarding simultaneous susceptibility, our results showed that in the group of women tested 2015–2018, patients originating from rural areas had a lower susceptibility rate than those from urban areas. In the group of women tested 2008–2010, no statistically significant difference was found between women from rural and urban areas regarding the susceptibility against two or three pathogens. Our results are in line with data from other countries (Mexico and Argentina) which mentioned a higher *Toxoplasma gondii* seroprevalence [20,21] in rural compared to urban areas or a higher CMV seroprevalence of women with a lower socioeconomic status [22].

Our large-scale study gave us the possibility to compare the simultaneous seroprevalence against the TORCH agents in women of childbearing age between the two periods: 2008–2010 and 2015–2018. We performed the comparison within the entire group of women or in detail, in the group originating in urban and rural areas. In the entire group of women, we observed a statistically extremely significant decrease in simultaneous seroprevalence to all combinations of two or three pathogens. Additionally, a statistically significant decrease was observed in women from urban areas, too. On the other hand, in women from rural areas, although the decreasing trend was present, it was statistically significant only for the simultaneous seroprevalence to *Toxoplasma gondii* and CMV, *Toxoplasma gondii* and rubella virus and to all the three agents, while for the simultaneous seroprevalence to CMV and rubella virus the descending trend was not statistically significant. This trend could be explained probably by an improvement in hygienic conditions with the passing of time. A similar descending evolution was reported for *Toxoplasma gondii* seroprevalence in the USA and for CMV seroprevalence in Croatia [23,24].

Since the women susceptible to infection with an agent are represented by the non-immunized ones, we studied the subgroups of women simultaneous seronegative to the three agents along the decade and according to the place of residence. Since in the group tested between 2008 and 2010 we did not find any patient seronegative to all the three agents, a comparison with the corresponding women from group 2 was not possible. Interestingly, in group 2 (2015–2018) the percentage of women susceptible to all tested agents was 0.71% in the entire group, 0.9% in the urban and 0.2% in the rural subgroup. Regarding the simultaneous susceptibility to two pathogenic agents, we found an ascending trend within the group of women tested in the interval 2008–2010 to the ones tested 2015–2018 only in the entire group and in women from urban areas. In women with a rural place of residence, no statistically significant difference was found between the two groups: 2008–2010 vs. 2015–2018. Our study, which assesses the susceptibility to the most important TORCH agents, brings new data relevant to build-up strategies to improve knowledge and behavior to prevent congenital toxoplasmosis, CMV and rubella virus infections [25].

It is worth mentioning the significant increase in the rate of women simultaneously susceptible to *Toxoplasma gondii* and CMV [OR 2.14 (all); OR 2.40 (urban)], or CMV and rubella virus [OR 14.57 (all); OR 12.23 (urban)] from group 1 to group 2. Furthermore, the rate of women who are simultaneously immune to the three pathogens decreased between the two time periods studied (OR = 0.75, *p* < 0.0001). Our data showed both an increase in the percentage of cases simultaneously susceptible to the three agents and a decrease in the rate of protected cases. Given the adverse effects of all these infections on the fetus, if they occur during pregnancy, it is necessary to apply increased prevention measures among susceptible pregnant women. Informing women of childbearing age about these pathogens and how to prevent infections can help pregnant women to avoid acute infections during pregnancy and congenital transmission. Some studies have shown that pregnancy education programs have been associated with improved knowledge and behavior and reduced rates of *Toxoplasma gondii* infection [26].

## 5. Conclusions

The rate of simultaneous seropositivity to *Toxoplasma gondii*, CMV and rubella virus among Romanian women of childbearing age decreased statistically significantly between 2008–2010 and 2015–2018, and the susceptibility to infections increased. The presented results may be useful for planning preventive measures and screening programs in fertile women in our region.

## Figures and Tables

**Table 1:** Demographic features of participants.

		Group 1 *n*/Total (%)	Group 2 *n*/Total (%)
Area of residence	Urban	1054/1461 (72.1%)	3570/5500(64.9%)
	Rural	407/1461 (27.9%)	1930/5500(35.1%)
Age of participants		14–43 years	14–45 years
Year of birth		1966–1994	1970–2004

**Table 2:** IgG- and IgM-anti-*Toxoplasma gondii*, -CMV and -rubella virus seroprevalence: (2008–2010) and (2015–2018).

	*Toxoplasma gondii*		CMV		Rubella Virus	
Group	IgG+ *n*/% (95%CI)	IgM+ *n*/% (95%CI)	IgG+ *n*/% (95%CI)	IgM+ *n*/% (95%CI)	IgG+ *n/*% (95%CI)	IgM+ *n*/% (95%CI)
1 (2008–2010) *n* = 1461	639/43.7%) (41.2–46.3%)	12/0.8% (0.5–1.4%)	1384/94.7% (93.5–95.8%)	5/0.3% (0.1–0.8%)	1375/94.1% (92.8–95.2%)	7/0.5% (0.2–1%)
2 (2015–2018) *n* = 5500	2132/38.8% (37.5–40.1%)	58/1.1% (0.8–1.4%)	5012/91.1% (90.3–91.9%)	16/0.3% (0.2–0.5%)	5032/91.5% (90.7–92.2%)	15/0.3% (0.2–0.4%)
*p* value	*p* = 0.0006	*p* = 0.55	*p* < 0.0001	*p* = 0.78	*p* = 0.0009	*p* = 0.19
Odds ratio (95%CI)	0.81 (0.72–0.91)	1.28 (0.68–2.46)	0.57 (0.99–0.72)	0.84 (0.32–2.12)	0.67 (0.47–0.85)	0.56 (0.24–1.48)

**Table 3:** Prevalence of women with positive both IgG-and IgM- antibody titers.

	Group 1 *n*/% (95% CI)	Group 2 *n*/% (95%CI)	*p* Value OR (95%CI)
*Toxoplasma gondii*	11/0.8% (0.4–1.3)	56/1.0% (0.8–1.3)	*p* = 0.45 1.35 (0.72–2.51)
CMV	4/0.3% (0.1–0.7)	16/0.3% (0.2–0.5)	*p* > 0.99 1.06 (0.36–2.93)
Rubella virus	7/0.5% (0.2–1.0)	15/0.3% (0.2–0.4)	*p* = 0.19 0.56 (0.24–1.48)

**Table 4:** Rate of women with simultaneous positive IgG-anti-*Toxoplasma gondii,* -CMV and -rubella virus antibodies according to the place of residence (2008–2010 vs. 2015–2018).

	Group 1 (2008–2010)				Group 2 (2015–2018)				Group 1 vs. Group 2		
	Overall *n*/Total % (95%CI)	Urban *n*/Total % (95%CI)	Rural *n*/Total % (95%CI)	*p* Value OR (95%CI)	Overall *n*/Total % (95%CI)	Urban *n*/Total % (95%CI)	Rural *n*/Total % (95%CI)	*p* Value OR (95%CI)	Overall *p* Value OR (95%CI)	Urban *p* Value OR (95%CI)	Rural *p* Value OR (95%CI)
*T. gondii* + CMV +	605/1461 41.4% (38.9–44.0)	405/1054 38.4% (35.5–41.4)	200/407 49.1% (44.3–54.0)	*p* = 0.0002 1.54 (1.23–1.94)	1986/3570 36.1% (34.8–37.4)	1134/3570 31.8% (30.3–33.3)	852/1930 44.1% (41.9–46.4)	*p* < 0.0001 1.69 (1.51–1.90)	0.0002 0.79 (0.71–0.89)	<0.0001 0.74 (0.64–0.85)	0.07 0.81 (0.65–1.01)
*T. gondii* – CMV –	43/1461 2.9% (2.2–3.9)	36/1054 3.4% (2.5–4.7)	7/407 1.7% (0.8–3.5)	*p* = 0.11 0.49 (0.21–1.11)	336/5500 6.1% (5.5–6.8)	280/3570 7.8% (7.0–8.8)	56/1930 2.9% (2.2–3.7)	*p* < 0.0001 0.35 (0.26–0.47)	<0.0001 2.14 (1.55–2.96)	<0.0001 2.40 (1.70–3.43)	0.23 1.70 (0.81–3.83)
*T. gondii* + Rubella +	611/1461 41.8% (39.3–44.4)	405/1054 38.4% (35.5–41.4)	206/407 50.6% (45.8–55.4)	*p* < 0.0001 1.64 (1.30–2.06)	1962/5500 35.7% (34.4–36.9)	1149/3570 32.2% (30.7–33.7)	813/1930 42.1% (39.9–44.3)	*p* < 0.0001 1.53 (1.36–1.71)	<0.0001 0.77 (0.68–0.86)	0.0002 0.76 (0.66–0.87)	0.002 0.71 (0.57–0.88)
*T. gondii* – Rubella –	58/1461 4.0% (3.1–5.1)	39/1054 3.7% (2.7–5.0)	19/407 4.7% (3.0–7.2)	*p* = 0.45 1.27 (0.73–2.25)	292/5500 5.3% (4.7–5.9)	201/3570 5.6% (4.9–6.4)	91/1930 4.7% (3.9–5.8)	*p* = 0.16 0.82 (0.64–1.06)	0.003 1.35 (1.01–1.80)	0.01 1.55 (1.10–2.20)	>0.99 1.01 (0.61–1.69)
CMV + Rubella +	1299/1461 88.9% 87.2–90.4)	936/1054 88.8% (86.8–90.6)	363/407 89.2% (85.8–91.8)	*p* = 0.92 1.04 (0.72–1.48)	4597/5500 83.6% (82.6–84.5)	2925/3570 81.9% (80.6–83.2)	1672/1930 86.6% (85.0–88.1)	*p* < 0.0001 1.42 (1.22–3.34)	<0.0001 0.63 (0.47–0.75)	<0.0001 0.57 (0.46–1.41)	0.16 0.78 (0.56–1.1)
CMV – Rubella –	1/1461 0.1% NA	1/1054 0.1% (NA)	0/407 0.0%	NA	53/5500 1.0% (07–1.3)	41/3570 1.1%(0.8–1.6)	12/1930 0.6% (0.4–1.1)	*p* = 0.06 0.53 (0.27–1.03)	<0.0001 14.57 (2.66–144.3)	0.0003 12.23 2.24–124.9)	NA
*T. gondii* + CMV + Rubella +	578/1461 39.6% (37.1–42.1)	384/1054 36.4% (33.6–39.4)	194/407 47.7% (42.9–52.5)	*p* = 0.0001 1.58 (1.26–2.00)	1824/5500 33.2% (31.9–34.4)	1049/3570 29.4% (27.9–30.9)	775/1930 40.2% (38.0–42.4)	*p* < 0.0001 1.61 (1.43–1.81)	<0.0001 0.75 (0.67–0.85)	<0.0001 0.72 (0.62–0.84)	0.005 0.73 (0.59–0.91)
*T. gondii* – CMV – Rubella –	0	0	0	NA	39/5500 0.71% (0.5–1.00)	31/3570 0.9% (0.6–1.2)	8/3570 0.2% (0.1–0.4)	*p* = 0.0003 0.25 (0.12–0.55)	NA	NA	NA

CMV = cytomegalovirus; NA = not applicable.

## Data Availability

The datasets used and/or analyzed during the present study are available from the first author on reasonable request.
